# Dismantling task-sharing psychosocial interventions to personalize care for people affected by common mental disorders in poor resource settings

**DOI:** 10.1192/j.eurpsy.2025.809

**Published:** 2025-08-26

**Authors:** D. Papola, V. Patel, C. Barbui

**Affiliations:** 1 University of Verona, Verona, Italy; 2 Harvard Medical School, Boston, United States

## Abstract

**Introduction:**

The global burden of common mental disorders is high, particularly for migrants and people living in low-resource settings. Although psychosocial interventions delivered by locally available lay or community health workers are effective, the mechanisms of intervention response are poorly understood. One of the major barriers is that psychosocial interventions are delivered as complex, multi-component ‘packages of care’.

**Objectives:**

The aim of the project is to systematically review all randomized controlled trials (RCTs) that have tested the efficacy of task-sharing psychosocial interventions for the treatment of people suffering from common mental disorders (depression, anxiety, and related somatic complaints), to dismantle the intervention protocols, to create a taxonomy of active intervention components, and to re-evaluate their efficacy.

**Methods:**

This project uses a mixed methods approach. In the first phase (qualitative), intervention manuals are reviewed and components are extracted to create a component taxonomy. The components and manual files were transferred to Dedoose, a qualitative data analysis computer software package. An initial two manuals were reviewed by two coders who piloted the entire codebook and assessed inter-rater reliability; any code discrepancies were discussed with a senior author. The two coders independently coded the same manual and repeated until an 80% IRR was achieved. The two coders then divided 12 manuals and coded them separately. In the second phase (quantitative analysis), we will use component network meta-analysis (cNMA) methodology. The main advantage of cNMA is the ability to disentangle intervention components and examine their effectiveness separately or in different combinations. According to the additive cNMA model which we will implement, adding a component “c” to a composite intervention “X” will lead to an increase (or decrease) of the effect size by an amount only dependent on “c”, and not on “X”. We will denote the corresponding component specific incremental standard mean difference (iSMD) so that iSMDc = SMD(X+c) v. (X). Combining these component-specific iSMDs will allow the estimation of SMD between any two composite interventions.

**Results:**

The component taxonomy will be presented at the conference, along with a network of comparisons and a hierarchy of all intervention components expressed as iSMD, indicating the added benefit of adding a component to an intervention.

**Conclusions:**

By selecting the most effective components, it will be possible to outline a novel task-shifting psychosocial intervention to be tested in future RCTs for the benefit of people with common mental disorders living in low-resource settings. These findings will form the basis for further investigations in the field of precision medicine.

Funded by HORIZON-MSCA-2021-PF-01 under grant agreement N101061648.

**Disclosure of Interest:**

None Declared

